# Evaluation of robotic versus open partial pancreatoduodenectomy—study protocol for a randomised controlled pilot trial (EUROPA, DRKS00020407)

**DOI:** 10.1186/s13063-020-04933-8

**Published:** 2021-01-08

**Authors:** Rosa Klotz, Colette Dörr-Harim, Thomas Bruckner, Philipp Knebel, Markus K. Diener, Thilo Hackert, André L. Mihaljevic

**Affiliations:** 1 Department of General, Visceral and Transplantation Surgery, Heidelberg University Hospital, Im Neuenheimer Feld 110, 69120 Heidelberg, Germany; 2 The Study Center of the German Surgical Society (SDGC), Heidelberg University Hospital, Im Neuenheimer Feld 110, 69120 Heidelberg, Germany; 3 Institute of Medical Biometry and Informatics, University of Heidelberg, Heidelberg, Germany

**Keywords:** Pancreatoduodenectomy, Robotic surgical procedures, Postoperative complication, Cost-benefit analysis, Randomised controlled trial

## Abstract

**Background:**

Partial pancreatoduodenectomy (PD) is the indicated surgical procedure for a wide range of benign and malignant diseases of the pancreatic head and distal bile duct and offers the only potential cure for pancreatic head cancer. The current gold standard, open PD (OPD) performed via laparotomy, is associated with morbidity in around 40% of cases, even at specialised centres. Robotic PD (RPD) might offer a viable alternative to OPD and has been shown to be feasible. Encouraging perioperative results have been reported for RPD in a number of small, non-randomised studies. However, since those studies showed a considerable risk of bias, a thorough comparison of RPD with OPD is warranted.

**Methods:**

The EUROPA (EvalUation of RObotic partial PAncreatoduodenectomy) trial is designed as a randomised controlled unblinded exploratory surgical trial with two parallel study groups. A total of 80 patients scheduled for elective PD will be randomised after giving written informed consent. Patients with borderline or non-resectable carcinoma of the pancreatic head as defined by the National Comprehensive Cancer Network guidelines, distant metastases or an American Society of Anaesthesiologists (ASA) score > III will be excluded. The experimental intervention, RPD, will be compared with the control intervention, OPD. An intraoperative dropout of approximately eight patients per group is expected because they may receive another type of surgical procedure than planned. Overall, 64 patients need to be analysed. The primary endpoint of the trial is overall postoperative morbidity within 90 days after index operation, measured using the Comprehensive Complication Index (CCI). The secondary endpoints include the feasibility of recruitment and assessment of clinical, oncological and safety parameters and quality of life and cost-effectiveness.

**Discussion:**

The EUROPA trial is the first randomised controlled trial comparing RPD with OPD. Differences in postoperative morbidity will be evaluated to design a future multicentre confirmatory efficacy trial.

**Trial registration:**

German Clinical Trial Register DRKS00020407. Registered on 9 March 2020

## Background

By 2030, pancreatic cancer is expected to be the second leading cause of cancer-related deaths in the USA [1]. Each year, 610,000–915,000 quality-adjusted life-years are lost to pancreatic cancer in Europe [2], dramatically impairing the quality of life of patients and caregivers. Surgical resection offers the only chance of long-term survival. Partial pancreatoduodenectomy (PD) is the indicated surgical procedure for pancreatic head cancer and for a wide range of benign and other malignant diseases of the pancreas and distal bile duct.

Advances in surgical technique and perioperative care have decreased the perioperative mortality of pancreatic resection to below 5% at specialised high-volume centres. Perioperative morbidity, however, remains high, with rates around 40% [3]. Therefore, measures to reduce postoperative complications are urgently needed. Minimally invasive surgery has been shown to decrease postoperative morbidity in a wide range of surgical indications. Laparoscopic PD was introduced in 1994 [4] and robotic PD (RPD) in 2003 [5]. Several small, non-randomised and mostly retrospective and single-centre studies and case series have investigated RPD and compared it with open PD (OPD), with inconsistent results [6–14]. While the operating time was longer for RPD in most studies [6, 8, 9, 12, 15], comparable positive margin rates [11] and similar incidences of postoperative haemorrhage [10] were reported. While some studies found a higher number of harvested lymph nodes [9, 13], lower blood loss [7–9, 12, 13, 15] and fewer major complications for RPD [7], other studies reported the contrary, with higher margin positivity [6] and fewer harvested lymph nodes for RPD [6]. A meta-analysis of the limited data derived from a small number of mainly observational studies revealed the superiority of RPD for three efficacy outcomes: wound infection rate, margin positivity rate and length of hospital stay [16].

Overall, the study results show a positive trend in favour of the robotic approach. The evidence for equivalence or superiority of RPD is limited, however, due to the lack of a high-quality randomised controlled trial (RCT). Therefore, based on the current literature and in line with current IDEAL recommendations (stage 2b) [17, 18], an investigator-initiated exploratory RCT with standardised procedures, management and outcome definitions is needed before widespread adoption of RPD can be considered. Therefore, the EUROPA pilot trial will collect data on postoperative complications, safety, feasibility of recruitment and costs in order to design a large multicentre confirmatory clinical trial.

## Methods/design

### Trial rationale

This exploratory trial aims to compare the two different operative approaches, OPD and RPD, with regard to postoperative morbidity within 90 postoperative days (POD), measured using the Comprehensive Complication Index (CCI).

### Trial design

EUROPA is an investigator-initiated, exploratory, open-label RCT with two parallel study groups.

### Study registration, ethics and consent

The trial protocol was approved by the ethics committee of the University of Heidelberg (*Ethikkommission Medizinische Fakultät Heidelberg*; S-025/2020, 3 February 2020), and the trial was registered with the German Clinical Trial Register (DRKS), an approved primary registry in the WHO network (DRKS00020407, 9 March 2020; UTN U1111-1245-8931, 30 December 2019) before inclusion of the first patient. The trial will be conducted at the Department of General, Visceral and Transplantation Surgery, University Hospital Heidelberg, Germany, in accordance with the Good Clinical Practice (GCP) guidelines and the Declaration of Helsinki. More than 250 PDs are performed each year at the Department of General, Visceral and Transplantation Surgery, and it is certified as a centre of excellence for minimally invasive surgery by the German Society for General and Visceral Surgery (*Deutsche Gesellschaft für Allgemein- und Viszeralchirurgie*). Conservatively, five patients per month can be included in the trial. Thus, given the need to recruit 80 patients (see the “Sample size calculation” section), completion of recruitment is feasible within 18 months. The independent statistical analysis will be performed at the Institute of Medical Biometry and Informatics (IMBI) of the University of Heidelberg. All patient-related information is subject to medical confidentiality according to the European General Data Protection Regulation (*Datenschutzgrundverordnung*, DSGVO), the Federal Data Protection Act (*Bundesdatenschutzgesetz*) and the State Data Protection Act (*Landesdatenschutzgesetz*).

### Study population

All patients scheduled for elective PD will be screened consecutively for eligibility and will be informed about the EUROPA trial during a pretreatment visit or on the day of hospitalisation. Eligible for participation are all adult patients planned for elective PD for any indication who are judged suitable for both RPD and OPD by the treating pancreatic surgeon. Patients must provide written informed consent to the responsible study physician and must be able to understand the individual consequences of the clinical trial. The following exclusion criteria have been defined: (1) borderline or unresectable carcinoma of the pancreatic head as defined in the National Comprehensive Cancer Network guidelines [19], (2) distant metastases, (3) American Society of Anesthesiologists (ASA) score > 3, (4) participation in another interventional trial interfering with the intervention and outcome of this trial and (5) anticipated language problems or lack of compliance.

### Withdrawal criteria

Participants may withdraw from the trial at their own request at any time without giving reasons. If PD cannot be performed (e.g. because of technical irresectability or metastatic disease), the patient concerned will leave the trial early. The latter category will be included in the final report of the trial to ensure complete transparency.

### Interventions

In order to avoid the risk of learning-associated bias, only surgeons with sufficient proficiency will be allowed to perform the randomised interventions. Proficiency in the open group will be defined as having performed 40 or more OPDs, and in the robotic group, 40 or more RPDs. The requirement for a minimum of 40 RPDs is based on the results of research into learning curves for RPD [12, 20, 21].

In both groups, after exclusion of hepatic and peritoneal metastases, PD is performed by conventional resection and reconstruction with pancreato-jejunostomy, hepatico-jejunostomy and duodeno-jejunostomy/gastro-jejunostomy according to local standards [22].

Resection or preservation of the pylorus, the extent of lymphadenectomy, additional vascular resections, abdominal drain placement and abdominal wall closure at the end of the procedure are left to the surgeon’s discretion and will be documented in the electronic case report form (eCRF).

OPD, the current institutional standard procedure, will be performed as described before [22].

RPD was introduced at the Department of General, Visceral and Transplantation Surgery in November 2016, accompanied by a tutoring and proctoring programme. The procedure has been standardised and is performed as described in the Intuitive® surgical procedure guide, written by the members of our department [23]. Key components of the RPD procedure are defined and will be adhered to in this trial in order to provide consistent, reproducible results. The surgeon can decide to convert from RPD to OPD due to intraoperative circumstances or technical problems, and the reasons for conversion will be documented.

The same perioperative and postoperative standard operating procedures are in place for both interventions, and patients in both groups will be accommodated on the same wards to ensure standardised postoperative care.

### Assignment of intervention and randomisation

All consecutive patients will be screened, and reasons for non-inclusion must be documented in a screening list. In order to achieve comparable intervention groups for known and unknown risk factors, patients will be randomised before surgery using a centralised web-based tool (randomizer.at) provided by the Institute of Medical Informatics, Statistics and Documentation of the Medical University of Graz. Therefore, selection bias (biassed allocation to interventions) is minimised by sequence generation and allocation concealment. Randomisation will be conducted only by authorised trial personnel.

### Primary and secondary endpoints

The primary endpoint of the EUROPA trial is cumulative morbidity within 90 days after PD, assessed by means of the CCI. The CCI is based on the established Clavien–Dindo classification of postoperative complications, which has gained widespread acceptance [24]. The Clavien–Dindo classification grades postoperative complications according to their sequelae, ranging from grade I (any deviation from the normal postoperative course without the need for pharmacological treatment) to grade V (death). The CCI score ranges from 0 (no complication of any kind during the postoperative course) to 100 (death of patient). The CCI permits objective assessment of cumulative postoperative morbidity even in an unblinded study setting. The CCI was developed jointly by surgeons and patients and considers the patient’s perspective as well as objective parameters of surgical effectiveness [25]. It is validated for the pancreatic surgical population and a difference of 10 is regarded as clinically relevant [26].

The secondary endpoints cover important oncological, clinical, economic and patient-relevant outcomes (Table 1).

**Table 1:** Definition of secondary endpoints

Secondary endpoint	Definition
General outcomes	
Feasibility of recruitment	Recruitment goal of *n* = 80 patients within 18 months.
Costs (€)	Procedure-related costs and all inpatient hospital costs up to POD 90.
Intraoperative outcomes	
Duration of surgery (min)	RPD: from the start of positioning of the robot to the end of skin closure. OPD: from the beginning of skin incision to the end of skin closure.
Blood loss (ml)	As recorded in the anaesthesiology report.
Serious intraoperative complications	Any untoward medical/surgical occurrence that results in death, is life-threatening, requires prolongation of existing hospitalisation or results in persistent or significant disability/incapacity. Intraoperative in this context is defined as from the beginning of anaesthesia until the end of skin closure.
Conversion rate (%)	Conversion rate from robotic to open surgery.
Surgeon’s mental workload/stress	Self-evaluation according to the National Aeronautics and Space Administration Task Load Index [27] at the end of surgery.
Oncological outcomes	
Rate of complete margin clearance in patients with malignant tumours	a. Microscopically complete margin clearance > 0.1 cm margin clearance, R0 (CRM−). b. Microscopic margin clearance ≤ 0.1 cm, R0 (CRM+). c. Microscopic margin involvement (R1) resections according to the 8th edition of the UICC TNM classification.
Lymph nodes in patients with malignant tumours	a. Number of lymph nodes resected. b. Number of tumour-positive lymph nodes.
Postoperative outcomes	
Mortality (%)	From the day of surgery until postoperative day 90.
Quality of recovery	Measured via the quality of recovery questionnaire QoR-15 [28], assessed at baseline and on POD 4.
Time to functional recovery (days)	Up to postoperative day 90; defined as: independently mobile at the preoperative level, sufficient pain control with oral medication alone, ability to maintain sufficient (i.e. > 50%) daily required caloric intake by mouth, no intravenous fluid administration and no signs of infection [29].
Total length of intensive care unit stay (days)	From the day of index operation up to postoperative day 90.
Length of hospital stay (days)	From the day of index operation up to the day of discharge.
Rate of superficial and deep surgical site infections (SSIs)	As defined by the Centres of Disease Control and Prevention (CDC) within 30 days [30]. Organ-space SSIs are excluded from this measurement as they are independent of surgical access, but rather depend on the underlying surgery. Consequently, organ-space SSIs will be recorded in the overall complication rate if applicable.
Pancreas-specific complications	Rate and severity within 90 days of: a. Postoperative pancreatic fistula as defined by the ISGPS [31]. b. Postpancreatectomy haemorrhage as defined by the ISGPS [32]. c. Delayed gastric emptying as defined by the ISGPS [33]. d. Biliary leak as defined by the ISGLS [34]. e. Chyle leak/lymphatic fistula as defined by the ISGPS [35].
Non-surgical re-interventions	Number of non-surgical re-interventions within 90 days after PD (e.g. image-guided drain placement, angiography with stenting/other interventions, endoscopy).
Re-operations	Number of re-operations within 90 days after PD.
Hospital re-admissions	Number of hospital re-admissions within 90 days after PD.
Pain	Pain scores at rest and during movement according to the Numeric Rating Scale (NRS) on POD 2 and 4.
Health-related quality of life (HRQoL)	Measured by the SF-36 at baseline, 30 and 90 days after index operation [36].

### Postoperative data collection and blinding

Prespecified study visits are carried out by clinical investigators and study nurses from the clinical study centre to collect information on the primary and secondary outcome parameters and to identify any postoperative complications.

Blinding of participants, research assistants, operating surgeons, data collectors and outcome assessors to the treatment allocation is not feasible. Postoperative blinding of patients, e.g. by the use of large dressings, is barely possible, since unblinding would occur when wound dressings were changed prior to the assessment of the primary endpoint. Furthermore, given the objective nature of the primary endpoint (morbidity according to the Clavien–Dindo classification), complex blinding interventions are not justified. In addition, numerous endpoints (economic parameters, safety endpoints, etc.) are independent of blinding. However, some secondary endpoints may be affected by the lack of blinding, e.g. pain or patient-reported outcomes such as quality of life or quality of recovery. Outcome assessments will be conducted by trained study personnel who are not part of the surgical or ward team, to guarantee objectivity. Data analysts will be blinded to the intervention.

### Description of trial visits

During the screening visit (1–14 days before surgery), inclusion and exclusion criteria are assessed (Table 2). After the patient has given informed consent, the demographic and baseline data, the ASA score, the updated Charlson Comorbidity Index [37], the health-related quality of life as measured by SF-36 [36], the quality of recovery as established using questionnaire QoR-15 [28] and the pancreas-specific medical history (indication for surgery, pre-existing diabetes mellitus, pre-existing exocrine insufficiency, neoadjuvant treatment, preoperative cholestasis, preoperative biliary drainage) are assessed and documented. Visit 2, including assessment of intraoperative and perioperative parameters as well as serious intraoperative complications, takes place on the day of surgery. Also documented during visit 2 are the type of pancreatic resection, extent of lymphadenectomy, additional organ resections, texture of the pancreas, size of the pancreatic duct at the transection site and drain insertion. Four inpatient follow-up visits on postoperative days (POD) 4, 8 and 12 and the day of discharge will be performed in person, and two telephone follow-up visits 30 and 90 days after surgery will be conducted to evaluate the primary and secondary outcome parameters (see Table 1). If a patient is discharged before POD 12, visit 5 can be omitted.

**Table 2:** Trial visits

Visit	1	2	3	4	5	6	7	8
	1–14 days before surgery	Day of surgery (day 0)	POD 4	POD 8	POD 12	Day of hospital discharge	POD 30	POD 90
	Outpatient/inpatient	Inpatient					Inpatient/telephone	
Eligibility criteria	X							
Informed consent	X							
Baseline demographics and clinical data	X							
Randomisation	X							
Quality of life questionnaire (SF-36)	X						X	X
Quality of recovery (QoR-15)	X		X					
Intervention		X						
Serious intraoperative complications		X						
Intraoperative secondary endpoints (e.g. operation time, blood loss, conversion rate, resection status)		X						
Surgeon’s mental workload/stress		X						
Assessment of primary endpoint (CCI)			X	X	X	X	X	X
Postoperative secondary endpoints (e.g. re-interventions, pain, time to functional recovery)			X*	X	X	X	X	X
Procedure-related costs, all inpatient hospital costs								X

*POD* postoperative day

*Pain needs to be assessed on POD 2 and 4

### Statistics

#### Sample size calculation

**Fig. 1:**
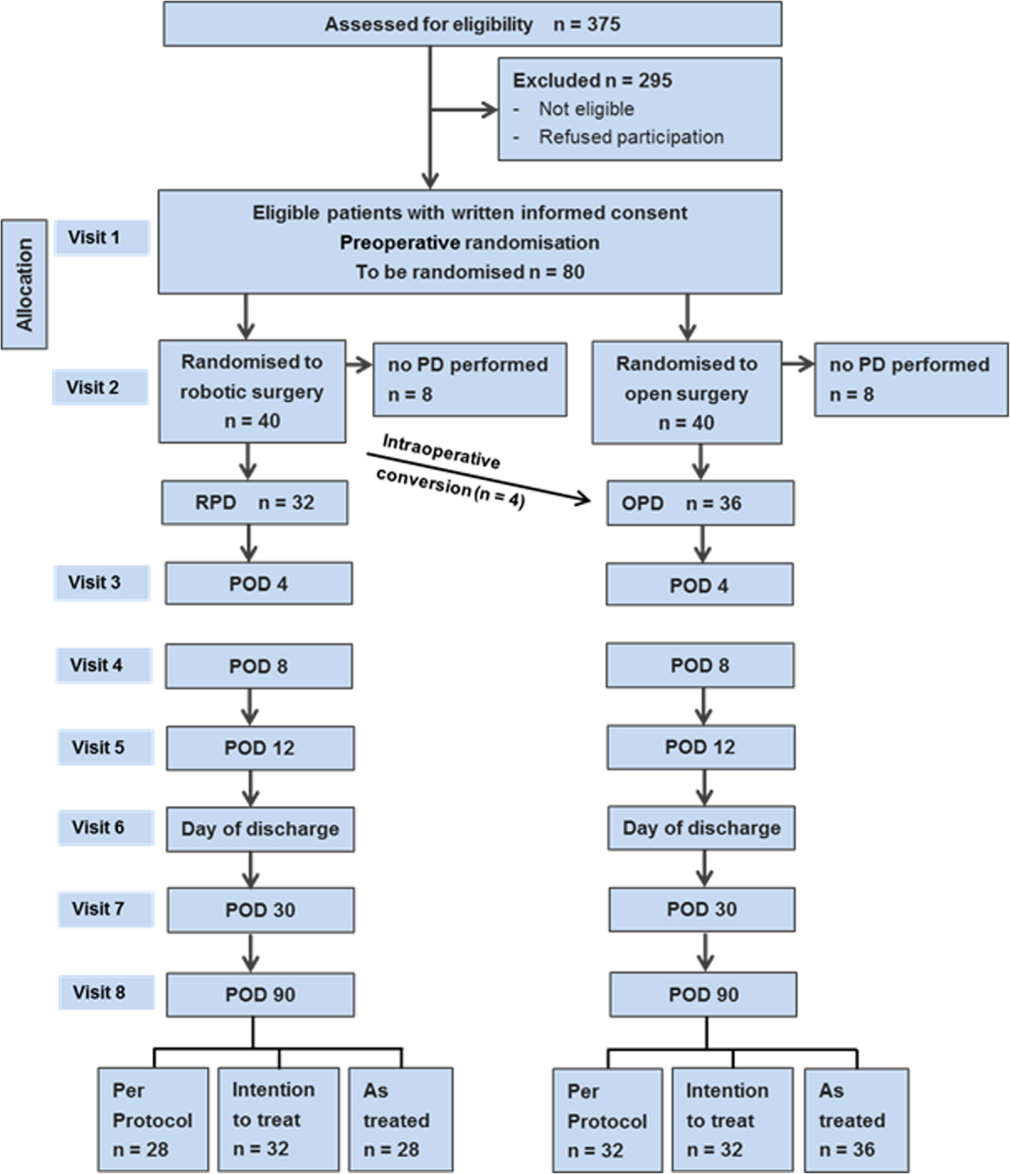
Flow chart of the EUROPA trial. POD, postoperative day; PD, partial pancreatoduodenectomy

Given that EUROPA is an exploratory trial, no formal sample size calculation was performed. However, a clinically relevant mean difference for the CCI is 10 [25, 26] while the mean standard deviation is approximately 20 in major abdominal surgery [38]. Including 64 patients (32 per group) in the analysis, this effect size could be estimated with a 95% confidence interval (CI) of [3.07, 16.93]. Considering that approximately eight patients per group will undergo neither RPD nor OPD due to inoperability or indication for another type of surgery (ascertained during the exploratory stage of the operation), a total of 80 patients will need to be allocated to the trial (Fig. 1).

#### Analysis variables and statistical methods

The primary endpoint is the CCI within 90 days after the intervention. As a primary result, the 95% CI for the difference in the means between the two groups will be reported. Since the CCI is approximately normally distributed [25], the 95% CI can be computed by means of the normal distribution. Further descriptive measures are reported in terms of mean and standard deviation.

Data analysis will be based on three analysis sets. The intention-to-treat (ITT) set comprises all patients in the group to which they were randomised (converted patients remain in the RPD group) and serves as the primary analysis set. Patients that receive an operation other than PD or total pancreatectomy (explorative laparotomy, diagnostic laparoscopy, etc.) are excluded from further analysis and proceed directly to the end-of-study visit (V8). The per-protocol (PP) set consists of all patients treated per protocol without major protocol violation and without conversions. No missing data will be imputed in the PP set. In addition, the as-treated set will be analysed, which considers the patients in the group in which they were finally treated (i.e. converted patients in the OPD group). There is no evidence that the converted patients will have a higher postoperative CCI than those patients randomised to and remaining in the OPD group. Therefore, and due to the exploratory character of this trial, the as-treated set is an important complement to the ITT and PP sets in this trial.

The primary endpoint is expected to be missing for a small number of patients, since it is objective and short term. In these cases, the primary endpoint is imputed using mean imputation based on the treatment group. As sensitivity analyses, worst-case and best-case scenarios are investigated. Missing values of the primary endpoint will be imputed using the lowest and the highest value of the corresponding treatment group.

The secondary endpoints are described as mean values along with standard deviations, median values, quartiles, minimum and maximum for continuous endpoints, and relative and absolute frequencies for categorical endpoints, stratified for treatment groups. Effect sizes (mean difference for continuous data, Hodges–Lehman estimator for scores and risk difference for binary data) including 95% CIs will be reported. The safety analysis includes calculation of frequencies and rates of both minor (Clavien–Dindo class I and II) and major complications (Clavien–Dindo class III to V) together with 95% CIs. Statistical methods will be used to assess the quality of data and the homogeneity of the intervention groups.

Details of statistical analyses as well as the handling of potential data problems will be defined in a statistical analysis plan (SAP) prior to database closure.

All analyses are exploratory, having only descriptive character, and will be conducted using SAS version 9.4 or higher.

#### Data management

An eCRF will be used for data collection (attached to this protocol as supplementary material). To ensure a safe and secure environment for the data acquired, data transmission is encrypted with secure socket layer (SSL) technology. All changes to data are logged with a computerised timestamp in an audit trail. All data will be pseudonymised. To guarantee high data quality, the data validation rules will be defined in a data validation plan. The completeness, validity and plausibility of data will be checked at the time of data entry (edit checks) and by using validating programmes that will generate queries. The completed eCRF must be reviewed and signed by the investigator named in the trial protocol or by a designated sub-investigator. The investigator or the designated representative is obliged to complete the eCRF as soon as possible after information is collected and to clarify or explain the queries.

Patients and surgeons will be identified solely by means of their individual identification code. Trial-specific documents will be stored in accordance with local data protection law and GCP guidelines and will be handled in strictest confidence. For the protection of these data, organisational procedures are implemented to prevent the distribution of data to unauthorised persons.

Once no further corrections are to be made, the database will be locked. All data management procedures will be conducted according to written defined standard operating procedures that guarantee efficient conduct complying with GCP. At the end of the study, the data will be transformed into different formats (e.g. csv files) for archiving purposes and to ensure future accessibility of the data.

#### Data monitoring

Quality assurance will be carried out following a risk-based strategy in cooperation of monitoring, data management and biostatistics. Independent monitoring includes clinical on-site visits and will follow standard operating procedures to ensure compliance with the trial protocol, the principles of the Declaration of Helsinki and GCP guidelines and data protection and other relevant legal aspects.

The primary endpoint, expressed in terms of the CCI, assesses safety. Therefore, adverse events are represented by minor complications (Clavien–Dindo class I and II) and serious adverse events are represented by major complications (Clavien–Dindo class III to V).

A Data and Safety Monitoring Board (DSMB) made up of independent experts and a patient representative will monitor the progress of the trial. DSMB members will receive a written safety report every 6 months and will advise on continuation, modification or termination of the trial.

The trial may be prematurely closed by the coordinating investigator in consultation with the steering committee and the DSMB. Factors that may necessitate termination of the trial include morbidity or complications that indicate a potential health hazard caused by the study treatment or relevant external evidence.

#### Methods for minimising bias

##### Minimising selection bias

The number of patients screened, the number included and the number analysed will be reported, and differences will be explained. The patient flow and the Consolidated Standards of Reporting Trials (CONSORT) flowchart will be reported in the final analysis.

##### Minimising attrition bias

The trial is registered with the German Clinical Trials Register (*Deutsches Register Klinischer Studien*; DRKS00020407). To avoid the risk of selective reporting, the trial protocol with full information about endpoints and a detailed explanation of the planned statistical analysis is hereby published according to the Standard Protocol Items: Recommendations for Interventional Trials (SPIRIT) statement. The SPIRIT Checklist is provided as supplementary file 1. Statistical measures such as imputation will be taken to minimise the risk of bias due to incomplete outcome data.

## Discussion

OPD represents the current clinical standard procedure, whereas RPD is performed only at a number of high-volume pancreatic centres. However, the technical aspects of the RPD procedure are standardised [39], and there is a publicly available surgical procedure guide for PD (Intuitive™; Intuitive Surgical Inc., Sunnyvale, CA, USA) [23], together with international exchanges, tutor programmes and centralised instruction/teaching. Early evaluations of a surgical innovation (whether an operation, invasive procedure or use of a medical device) face a common set of difficulties. Unfavourable outcomes in the initial period after the introduction of new surgical techniques are a well-known phenomenon and could endanger patient safety [40]. Therefore, the IDEAL recommendations propose a framework for the introduction, evaluation, assessment and follow-up of new surgical techniques using prospective study designs [17]. RCTs with a standardised technique provide the best evidence. The EUROPA trial is designed as a pragmatic trial, since the objective is to depict the effectiveness and safety of interventions in real-life routine practice conditions and produce generalisable results. The broad inclusion criteria and the low number of exclusion criteria represent the pragmatic nature of this trial and the aim to obtain results with high generalisability and high external validity.

The previous studies, mostly retrospective and performed at a single centre, may be affected by selection and attrition bias. Despite early promising results, the evidence for equivalence or superiority of RPD is restricted by the absence of RCTs. Accordingly, this exploratory RCT with clear standardisation of procedures, management and outcome definitions is highly warranted. RPD needs thorough investigator-initiated evaluation (stage 2b of the IDEAL recommendation) now, before widespread adoption of the technique can be considered. It has been shown that, as for pharmacological trials, an industry bias also exists in surgery: industry-funded trials are 5 times more likely to present positive outcomes than trials without industry funding [41]. Accordingly, early investigator-initiated trials with public funding are necessary to evaluate new surgical techniques.

No competing clinical trial has been identified in any of the following clinical databases: ClinicalTrials.gov, Current Controlled Trials, University Medical Information Network (UMIN) Clinical Trials Registry and German Clinical Trials Register (DRKS).

One RCT comparing minimally invasive surgery (either laparoscopic (*n* = 42 patients) or robotic (*n* = 5 patients)) with open surgery (Dutch Trial Register, NTR5689) was terminated early due to high mortality in the minimally invasive group [29]. However, RPD was underrepresented in this trial, and laparoscopic and robotic PD are not equivalent in terms of risks, complications or costs. Collective evaluation of the two strategies would distort a number of critical endpoints and is not meaningful, as the robotic approach clearly differs from laparoscopic PD. Accordingly, a valid conclusion about RPD cannot be drawn from this trial due to the very low proportion of patients that underwent RPD.

The available data on overall morbidity rates are heterogeneous (RPD 25–76% and OPD 40–75%) [7–9, 14, 21]. Study populations and definitions of endpoints were heterogeneous or were not sufficiently defined in previous studies. In addition, the learning curve of participating surgeons was addressed in only some of the studies [6, 12]. Furthermore, data on long-term results and cost-effectiveness are lacking.

The current vivid discussion on robotic surgery addresses cost-effectiveness issues in most cases. However, sound-randomised evidence for surgical and oncological effectiveness as well as safety are rarely available, which hampers these scientific discussions. This trial is the first step to establish high-level evidence on efficacy and safety for RPD as a required basis for a future assessment of cost-effectiveness.

If the results of this pilot trial are promising, a high-quality multicentre confirmatory superiority trial comparing RPD and OPD in terms of the primary endpoint CCI will be planned. To this end, several aspects need to be elucidated in our exploratory trial: (a) It is unclear whether and how patients planned for PD are willing to undergo randomisation between RPD and OPD. Furthermore, as RPD is associated with considerable organisational challenges (restriction to specialised operation rooms, capacity, etc.), more data need to be collected to enable sound planning of recruitment in a confirmatory trial. (b) The results and subgroup analyses from our exploratory trial will help to define the target population of a future confirmatory trial. (c) High-quality data on postoperative morbidity will be collected in our trial to enable sound sample size calculation.

A confirmatory trial of this nature will answer the question of whether the higher costs of RPD are justified by superiority in terms of overall morbidity and/or oncological outcome. Such a trial would also allow evaluation of long-term outcomes, including overall and disease-free survival, in pancreatic cancer patients. In the confirmatory trial, stratification for the centre and patient subgroup will be performed and centre effects will be evaluated. This trial will indicate whether RPD should be preferred and in which patients.

### Trial status

Recruitment of the EUROPA trial started in June 2020. Recruitment is expected to be complete in 2022. The current version of the protocol is version 1.1, finalised on 2 February 2020.

## Supplementary Information

**Additional file 1: Supplementary file 1.** Clavien–Dindo classification of surgical complications [18].

**Additional file 2.** Electronic case report form.

## Data Availability

After completion of the trial, the data obtained will be summarised and analysed according to this protocol and then published in a peer-reviewed journal. Furthermore, dissemination will be carried out via online media in lay language and by contacting appropriate patient counselling groups to ensure accessibility to any healthcare professional, participant or member of the public. The full study protocol is available upon request. An anonymised minimal data set laying out the results of the trial will be made available upon publication of the final results as a supplement in line with national and international data protection rules. The statistical analysis plan will be available upon request after the publication of the final results.

## Abbreviations

ASA: American Society of Anesthesiologists
CCI: Comprehensive Complication Index
eCRF: Electronic case report form
IMBI: Institute for Medical Biometry and Informatics
KSC: Clinical trial centre surgery (*Klinisches Studienzentrum Chirurgie*)
OPD: Open pancreatoduodenectomy
PD: Pancreatoduodenectomy
POD: Postoperative day
RCT: Randomised controlled trial
RPD: Robotic pancreatoduodenectomy
SSI: Surgical site infection
