# Supplementary material for: Evaluation of robotic versus open partial pancreatoduodenectomy—study protocol for a randomised controlled pilot trial (EUROPA, DRKS00020407)
Source: Trials. 2021 Jan 8;22:40. doi: 10.1186/s13063-020-04933-8 (PMC7796523; doi:10.1186/s13063-020-04933-8)
Supplement: Supplementary file 2 — Additional file 2. Electronic case report form. [file 13063_2020_4933_MOESM2_ESM.pdf]

Visit 1 Screening		
**Variable**	**Type**	**Value**
Centre	(automatic REDCap data access group variable)	(name of data access group)
Screening no.<br>(must provide value)	integer	
Date of screening visit	date	
**Demographic data**		
Age (years)	integer	(xxx)
Sex	radio	male<br>female<br>diverse
Height (cm)	integer	(xxx)
Weight (kg)	integer	(xxx)
**Baseline clinical data**		
Smoking	radio	Current smoker<br>Former smoker<br>Non-smoker
Alcohol consumption	radio	Yes, often (daily, almost daily)<br>Yes, occasionally (approximately once a week)<br>Yes, rarely (less than once a week)<br>No current alcohol consumption, but drank in the past<br>No, never drank alcohol
American Society of Anesthesiologists (ASA) classification	radio	ASA I<br>ASA II<br>ASA III<br>ASA IV
Previous abdominal surgeries	radio	Yes<br>No
if yes: Number of previous abdominal surgeries	integer	(x)
if yes: Number of previous minimally invasive abdominal surgeries	integer	(x)
Current (within 14 days before surgery) medication	radio	Yes<br>No
if yes: Glucocorticoids	radio	Yes<br>No
Immunosuppressives	radio	Yes<br>No
Analgetics	radio	Yes<br>No
Octreotide, Somatostatin(-analogues)	radio	Yes<br>No
Anticoagulants	radio	Yes<br>No
**Pancreas specific medical history**		
Indication for surgery	radio	Malignant<br>Benign

if malignant: (Suspected) type of cancer	radio	Pancreatic ductal adenocarcinoma<br>Neuroendocrine tumour (NET)<br>Distal cholangiocarcinoma<br>Ampullary cancer ("Papillenkarzinom")<br>Serous cystic neoplasia (SCN)<br>Mucinous cystic neoplasia (MCN)<br>Intraductal papillary-mucinous carcinoma (IPMN-carcinoma)<br>Acinar cell carcinoma<br>Solid-pseudopapillary neoplasia (SPN)<br>Other
if other: Please specify other type of cancer	text	
if benign: Type of disease	Radio	Chronic pancreatitis<br>Autoimmune pancreatitis<br>Intraductal papillary mucinous neoplasm (IPMN)<br>Other
if other: Please specify other disease	text	
Preexisting diabetes mellitus	radio	Yes<br>No
Preexisting exocrine insufficiency with pancreatic enzyme supplementation	radio	Yes<br>No
if indication=malignant:<br>Status post neoadjuvant treatment	radio	Yes<br>No
Preoperative cholestasis	radio	Yes<br>No
if yes: Preoperative total bilirubin level (mg/dl)	number	(xx.x)
if yes: Jaundice	radio	Yes<br>No
Preoperative biliary drainage	radio	Yes<br>No
if yes: Endoscopic drainage	radio	Yes<br>No
Percutaneous drainage	radio	Yes<br>No
Operative biliary drainage	radio	Yes<br>No
**Charlson Comorbidity Index (revised version)**		
Congestive heart failure	radio	Yes (2 Pts.)<br>No
Dementia	radio	Yes (2 Pts.)<br>No
Chronic pulmonary disease	radio	Yes (1 Pts.)<br>No
Rheumatologic disease	radio	Yes (1 Pts.)<br>No
Mild liver disease	radio	Yes (2 Pts.)<br>No
Diabetes with chronic complications	radio	Yes (1 Pts.)<br>No
Hemiplegia or paraplegia	radio	Yes (2 Pts.)<br>No

Renal disease	radio	Yes (1 Pt.)<br>No
Any malignancy, including leukemia and lymphoma	radio	Yes (2 Pts.)<br>No
Moderate or severe liver disease	radio	Yes (4 Pts.)<br>No
Metastatic solid tumor	radio	Yes (6 Pts.)<br>No
AIDS/HIV	radio	Yes (4 Pts.)<br>No
**Inclusion criteria**		
Planned elective partial pancreatoduodenectomy for any indication	radio	Yes<br>No
Patient equally suitable for both RPD and OPD as judged by the treating pancreatic surgeon	radio	Yes<br>No
Age ≥ 18 years	radio	Yes<br>No
Ability of subject to understand character and individual consequences of the clinical trial	radio	Yes<br>No
Written informed consent	radio	Yes<br>No
if yes: Date of written informed consent	date	
**Exclusion criteria**		
Borderline or non-resectable carcinomas of the pancreatic head as defined by the National Comprehensive Cancer Network guidelines	radio	Yes<br>No
Distant metastases	radio	Yes<br>No
American Society of Anaesthesiologists (ASA) Score > 3	radio	Yes<br>No
Participation in another interventional trial with interference of intervention and outcome of this trial	radio	Yes<br>No
Expected lack of compliance or language problems	radio	Yes<br>No
**Patient reported outcome**		
SF-36 completed	radio	Yes<br>No
QoR-15 completed	radio	Yes<br>No
**Randomization**		
Randomization number<br>(must provide value)	integer	(xxx)
Treatment group<br>(must provide value)	radio	Robotic partial pancreatoduodenectomy (RPD)<br>Open partial pancreatoduodenectomy (OPD)

Visit 2 Surgery		
Variable	Type	Value
Date of surgery	date	
**Surgeon's expertise**		
Surgeon has performed ≥ 40 RPDs	radio	Yes<br>No
Surgeon has performed ≥ 40 OPD	radio	Yes<br>No
**Operation details**		
Procedure started as<br>(must provide value)	radio	RPD<br>OPD
if procedure started as = RPD:<br>start of positioning of the robot (hh:mm)	time	
if procedure started as = OPD:<br>Start of skin incision (hh:mm)	time	
End of skin closure (hh:mm)	time	
If procedure started= RPD:<br>Intraoperative conversion to open resection	radio	Yes<br>No
if yes:<br>Reason	radio	Technical difficulties<br>Underlying disease<br>Other
if reason = other:<br>Specification of other reason	text	
Intraoperative blood loss (according to the anaesthesiology report) (ml)	integer	(xxxxx)
Blood transfusion	radio	Yes<br>No
if yes: Number of PRBC	integer	(x)
**Type of pancreatic resection**		
Degree of pancreatic resection	radio	Partial pancreatoduodenectomy<br>Total pancreatectomy<br>Other<br>No pancreatoduodenectomy performed (end of study)
if degree = other:<br>Specification of degree of pancreatic resection	text	
**following items will only be asked if degree of pancreatic resection is not answered 'no pancreatoduodenectomy performed'**		
Degree of stomach resection	radio	Pylorus-preserving<br>Pylorus resecting<br>Classic (1/3 resection of stomach)<br>(Sub)total gastrectomy<br>Other
Degree of SMA dissection	radio	Inoue level 1<br>Inoue level 2<br>Inoue level 3
Triangle procedure performed	radio	Yes<br>No

Interaortocaval or paraaortal lymphadenectomy performed	radio	Yes<br>No
Venous resection performed	radio	Yes<br>No
Arterial resection performed	radio	Yes<br>No
Resection of additional organs performed beyond PD	radio	Yes<br>No
Texture of pancreas	radio	Soft<br>Medium<br>Hard
Size of the pancreatic duct at transection site	radio	≤ 3mm<br>>3mm
Drain insertion	radio	Yes<br>No
Surgical reconstruction performed during index surgery	radio	Yes<br>No
if no: Date of reconstruction operation	date	
if no: Start of reconstruction operation (hh:mm)	time	
if no: End of reconstruction operation (hh:mm)	time	
**Intraoperative serious complications (for all patients)**		
Intraoperative serious complication	radio	Yes<br>No
if yes: Specification of serious complication	text	
Seriousness	radio	Results in death<br>Is life-threatening<br>Requires prolongation of existing hospitalisation<br>Results in persistent or significant disability or incapacity
**following items will only be asked if degree of pancreatic resection is not answered 'no pancreatoduodenectomy performed'**		
**NASA Task Load Index**		
NASA Task Load Index completed	radio	Yes<br>No
**Protocol violations**		
Any protocol violation	radio	Yes<br>No
if yes: Specification of protocol violation	text	

if degree of pancreatic resection=no pancreatoduodenectomy performed: **Signature (only accessible for users with role 'Investigator')**

Variable	Type	Value
I confirm that all data entered in the eCRF is complete and accurate to the best of my knowledge	date	(date of confirmation should be entered)
Investigator's signature	signature file	

if degree of pancreatic resection is not answered 'no pancreatoduodenectomy performed':<br>**Visit 2 NASA Task Load Index**		
Variable	Type	Value
How mentally demanding was the task? (0-20)	integer	(xx)
How physically demanding was the task? (0-20)	integer	(xx)
How hurried or rushed was the pace of the task? (0-20)	integer	(xx)
How successful were you in accomplishing what you were asked to do? (0-20)	integer	(xx)
How hard did you have to work to accomplish your level of performance? (0-20)	integer	(xx)
How insecure, discouraged, irritated, stressed, and annoyed were you? (0-20)	integer	(xx)

if degree of pancreatic resection is not answered 'no pancreatoduodenectomy performed': **Visit 2 Histopathology**		
Variable	Type	Value
Histopathological assessment	radio	Malignant<br>Benign neoplastic lesion<br>Benign non-neoplastic lesion
If malignant:<br>Pancreatic cancer subtype according to the current classification of the WHO	radio	Pancreatic ductal adenocarcinoma<br>Intrapancreatic cholangiocarcinoma<br>Ampullary cancer ("Papillenkarzinom")<br>Serous cystadenocarcinoma<br>Mucinous cystadenocarcinoma<br>Intraductal papillary-mucinous carcinoma<br>Acinar cell carcinoma<br>Solid-pseudopapillary neoplasia<br>Neuroendocrine tumour
if malignant:<br>Resection status	radio	Microscopically complete margin clearance >0.1 cm margin clearance, R0 CRM-<br>Microscopic margin clearance ≤0.1cm, R0 CRM+<br>Microscopic margin involvement, R1
if malignant:<br>Number of resected lymph nodes	integer	(xx)
if malignant:<br>Number of tumour positive lymph nodes	integer	(xx)
if malignant:<br>Distant metastases	radio	M0<br>M1<br>Mx (cM, uM or pM)
If benign neoplastic lesion:<br>Benign neoplastic lesion	radio	Intraductal papillary mucinous neoplasm (IPMN)<br>Serous cystic neoplasm (SCN)<br>Mucinous cystic neoplasm (MCN)<br>Other

if other: Specification of other neoplastic lesion	text	
If benign non-neoplastic lesion: Specification of lesion	radio	Pseudocysts<br>Chronic pancreatitis<br>Autoimmune pancreatitis<br>Other
if other: Specification of other non-neoplastic lesion	text	

if degree of pancreatic resection is not answered 'no pancreatoduodenectomy performed':<br>**Visit 3 POD 4, Visit 4 POD 8, Visit 5 POD 12, Visit 6 day of discharge**		
Variable	Type	Value
if visit= visit 3 or visit 4 or visit 5:<br>Date of visit	date	
if visit= visit 6:<br>Date of discharge	date	
**Functional recovery**<br>Please check items for functional recovery on global form 'Functional recovery', please complete it latest on last visit		
**if visit=visit 6: Intensive care unit (ICU) stay**		
if visit=visit 6: ICU stay from index surgery until discharge	radio	Yes<br>No
if yes: number of days on ICU	integer	(xxx)
**if visit= visit 3: Pain assessment**		
**Pain assessment on POD2**		
if visit= visit 3: Pain at rest (NRS 0-10)	integer	(xx)
if visit= visit 3: Pain during movement (NRS 0-10)	integer	(xx)
**Pain assessment on POD4**		
if visit= visit 3: Pain at rest (NRS 0-10)	integer	(xx)
if visit= visit 3: Pain during movement (NRS 0-10)	integer	(xx)
**Pancreas-specific complications since last visit**		
Postoperative pancreatic fistula as defined by the ISGPS	radio	No POPF<br>Biochemical Leak<br>Grade B<br>Grade C
Postpancreatectomy haemorrhage as defined by the ISGPS	radio	No haemorrhage<br>Grade A<br>Grade B<br>Grade C
Delayed gastric emptying as defined by the ISGPS	radio	no DGE<br>Grade A<br>Grade B<br>Grade C

Biliary leak as defined by the ISGLS	radio	No leak<br>Grade A<br>Grade B<br>Grade C
Chyle leak / lymphatic fistula as defined by the ISGPS	radio	No chyle leak / no lymphatic fistula<br>Grade A<br>Grade B<br>Grade C
**Other complications since last visit**		
Surgical site infection according to CDC	radio	No SSI<br>Superficial SSI<br>Deep SSI
Other complications	radio	Yes<br>No
in case of any complication (one of defined complication at least Grade A / biochemical leak or SSI or other complication = yes):	descriptive	Please enter complication(s) in eForm 'Complications'
**Non-surgical interventions since last visit**		
Any non-surgical intervention(s) since last visit<br>(CT guided drain placement / Angiography with stenting / Endoscopy / Other)	radio	Yes<br>No
if yes:	descriptive	Please enter every non-surgical intervention in eForm 'Non-surgical intervention' and reason(s) for intervention in eForm 'Complication'
**Re-operation since last visit**		
Re-operation since last visit	radio	Yes<br>No
if yes:	descriptive	Please enter re-operation(s) in eForm 'Reoperation' and reason(s) for re-operation in eForm 'Complication'
**If visit=visit 3: Patient reported outcome**		
QoR-15 completed	radio	Yes<br>No
**Any protocol violation since last visit**		
Any protocol violation	radio	Yes<br>No
if yes: Specification of protocol violation	text	

if degree of pancreatic resection is not answered 'no pancreatoduodenectomy performed':
Visit 7 POD 30, Visit 8 POD90/EoS

Variable	Type	Value
if visit= visit 7:<br>Please tick this box if visit was not performed	Checkbox	Not done
If 'not done' is ticked no further entry is possible for respective visit		

Study termination		
if visit= visit 8:<br>Was the trial terminated regularly for this patient	radio	Yes<br>No
if no: Date of premature trial termination /last contact	date	
if no: Reason for premature trial termination	radio	Patient died<br>Withdrawal of informed consent<br>Lost to follow-up<br>Other reason
if other reason: Specification of other reason for premature termination	text	
if death: Date of death	date	
if death: Cause of death	radio	Related to index surgery<br>Other<br>Unknown
If other: Specification of other cause of death	text	
if terminated regularly = yes or visit = v7:<br>Date of visit	date	
**Functional recovery**<br>Please check items for functional recovery on global form 'Functional recovery', please complete it latest on last visit		
**if visit=visit 8 and discharge date = missing (patient not discharged): Intensive care unit (ICU) stay**		
if visit=visit 8 and discharge date = missing: ICU stay from index surgery until discharge	radio	Yes<br>No
if yes: Number of days on ICU	integer	(xxx)
**Pancreas-specific complications since last visit**		
Postoperative pancreatic fistula as defined by the ISGPS	radio	No POPF<br>Biochemical Leak<br>Grade B<br>Grade C
Postpancreatectomy haemorrhage as defined by the ISGPS	radio	No haemorrhage<br>Grade A<br>Grade B<br>Grade C
Delayed gastric emptying as defined by the ISGPS	radio	no DGE<br>Grade A<br>Grade B<br>Grade C
Biliary leak as defined by the ISGLS	radio	No leak<br>Grade A<br>Grade B<br>Grade C
Chyle leak / lymphatic fistula as defined by the ISGPS	radio	No chyle leak / no lymphatic fistula<br>Grade A<br>Grade B<br>Grade C
**Other complications since last visit**		

if visit=visit 7:<br>Surgical site infection according to CDC	radio	No SSI<br>Superficial SSI<br>Deep SSI
Other complications	radio	Yes<br>No
in case of any complication (one of defined complication at least Grade A / biochemical leak or SSI or other complication = yes):	descriptive	Please enter complication(s) in eForm 'Complications'
**Non-surgical interventions since last visit**		
Any non-surgical intervention(s) since last visit<br>(CT guided drain placement / Angiography with stenting / Endoscopy / Other)	radio	Yes<br>No
if yes:	descriptive	Please enter every non-surgical intervention in eForm 'Non-surgical intervention' and reason(s) for intervention in eForm 'Complication'
**Re-operation since last visit**		
Re-operation since last visit	radio	Yes<br>No
if yes:	descriptive	Please enter re-operation(s) in eForm 'Reoperation' and reason(s) for re-operation in eForm 'Complication'
if visit 6 discharge date not missing: **Readmission to hospital since last visit**		
Readmission to hospital since last visit	radio	Yes<br>No
if yes:	descriptive	Please enter readmission(s) in eForm 'Readmission' and reason(s) for readmission in eForm 'Complication'
**if visit=8: Costs**		
Procedure related costs (Euro)	integer	(xxxxxx)
Overall inpatient costs (Euro)	integer	(xxxxxx)
**if visit=7 or visit=8 and trial terminated regularly=yes: Patient reported outcome**		
SF-36 completed	radio	Yes<br>No
**Any protocol violation since last visit**		
Any protocol violation	radio	Yes<br>No
if yes: Specification of protocol violation	text	

if degree of pancreatic resection is not answered 'no pancreatoduodenectomy performed':
Signature (only accessible for users with role 'Investigator')

Variable	Type	Value
I confirm that all data entered in the eCRF is complete and accurate to the best of my knowledge	date	(date of confirmation should be entered)
Investigator's signature	signature file	

Global Pages

if degree of pancreatic resection is not answered 'no pancreatoduodenectomy performed': **Functional recovery**		
Variable	Type	Value
**Functional recovery**		
Independently mobile at the preoperative level	radio	Yes<br>No
if yes: Date since when	date	
Sufficient pain control with oral pain medication	radio	Yes<br>No
if yes: Date since when	date	
Ability to maintain sufficient (i.e. >50%) daily required caloric intake by mouth	radio	Yes<br>No
if yes: Date since when	date	
No intravenous fluid administration	radio	Yes<br>No
if yes: Date since when	date	
No signs of infection	radio	Yes<br>No
if yes: Date since when	date	

if degree of pancreatic resection is not answered 'no pancreatoduodenectomy performed': **Complications (repeatable instrument)**		
Variable	Type	Value
Complication	text	
Grading according to Clavien-Dindo	radio	Grade I<br>Grade II<br>Grade IIIa<br>Grade IIIb<br>Grade IVa<br>Grade IVb<br>Grade V
Date of onset	date	
if grade not answered 'Grade V': Ongoing at time of hospital discharge	radio	Yes<br>No

if degree of pancreatic resection is not answered 'no pancreatoduodenectomy performed':<br>**Non-surgical intervention (repeatable instrument)**		
Variable	Type	Value
Type of non-surgical intervention	radio	CT guided drain placement<br>Angiography (with/without stenting/coiling)<br>Endoscopy<br>Other
Specification of other intervention	text	
Please enter reason for non-surgical intervention as complication in eForm 'Complications'		

if degree of pancreatic resection is not answered 'no pancreatoduodenectomy performed':**Re-operation (repeatable instrument)**		
Variable	Type	Value
Specification of re-operation	text	
Date of re-operation	date	
Please enter reason for re-operation as complication in eForm 'Complications'		

if degree of pancreatic resection is not answered 'no pancreatoduodenectomy performed':<br>**Readmission to hospital (repeatable instrument)**		
Variable	Type	Value
Date of admission to hospital	date	
Reason for readmission	radio	Related to index operation<br>Related to underlying index disease<br>Unrelated to index operation or underlying index disease
Intervention	checkbox	Re-operation<br>Non-surgical intervention<br>Medication change<br>Other
if other: Specification of other intervention	text	
if re-operation:	descriptive	Please enter re-operation(s) in eForm 'Reoperation' and reason(s) for re-operation in eForm 'Complication'
if non-surgical intervention:	descriptive	Please enter non-surgical intervention in eForm 'Non-surgical intervention' and reason(s) for intervention in eForm 'Complication'
Ongoing hospital stay at end of study	radio	yes<br>no
if no: End of re-hospitalisation	date	

Stay on ICU	radio	yes<br>no
if yes: Number of days on ICU	integer	(xx)

Patient Reported Outcome

**QoR-15 (Quality of Recovery) (visit 1, visit 3** only if degree of pancreatic resection is not answered 'no pancreatoduodenectomy performed'**)**		
Variable	Type	Value
Datum des Ausfüllens<br>*If exact date not known, please skip this field and enter year and month (if known) below*	date	
Falls Datum unvollständig angegeben, bitte Monat eintragen	dropdown	01<br>02<br>03<br>04<br>…<br>11<br>12
Falls Datum unvollständig angegeben, bitte Jahr eintragen	integer	
**Teil A**	descriptive	Wie haben Sie sich in den letzten 24 Stunden gefühlt?
1. Ich konnte problemlos durchatmen.	Radio matrix	0, zu keinem Zeitpunkt<br>1-9<br>10, Immer
2. Ich konnte das Essen genießen.	Radio matrix	0, zu keinem Zeitpunkt<br>1-9<br>10, Immer
3. Ich habe mich ausgeruht gefühlt.	Radio matrix	0, zu keinem Zeitpunkt<br>1-9<br>10, Immer
4. Ich konnte gut schlafen.	Radio matrix	0, zu keinem Zeitpunkt<br>1-9<br>10, Immer
5. Ich war in der Lage, mich selbst (ohne Hilfe) um meine persönliche Hygiene zu kümmern.	Radio matrix	0, zu keinem Zeitpunkt<br>1-9<br>10, Immer
6. Ich konnte mich mit meiner Familie und mit Freunden unterhalten.	Radio matrix	0, zu keinem Zeitpunkt<br>1-9<br>10, Immer
7. Unterstützung durch Klinikärzte und der Pflege	Radio matrix	0, zu keinem Zeitpunkt<br>1-9<br>10, Immer
8. Ich wäre in der Lage, zur Arbeit oder den normalen häuslichen Aktivitäten zurückzukehren.	Radio matrix	0, zu keinem Zeitpunkt<br>1-9<br>10, Immer
9. Ich habe mich angenehm gefühlt und hatte das Gefühl, Einfluss auf den Lauf der Dinge zu haben.	Radio matrix	0, zu keinem Zeitpunkt<br>1-9<br>10, Immer
10. Ich habe ein Gefühl des Wohlbefindens.	Radio matrix	0, zu keinem Zeitpunkt<br>1-9<br>10, Immer
**Teil B**	descriptive	Hatten Sie während der letzten 24 Stunden die folgenden Beschwerden?

11. Moderate Schmerzen	Radio matrix	10, zu keinem Zeitpunkt<br>9-1<br>0, Immer
12. Starke Schmerzen	Radio matrix	10, zu keinem Zeitpunkt<br>9-1<br>0, Immer
13. Übelkeit und Erbrechen	Radio matrix	10, zu keinem Zeitpunkt<br>9-1<br>0, Immer
14. Ich habe mir Sorgen gemacht und mich ängstlich gefühlt.	Radio matrix	10, zu keinem Zeitpunkt<br>9-1<br>0, Immer
15. Ich habe mich traurig und niedergeschlagen gefühlt.	Radio matrix	10, zu keinem Zeitpunkt<br>9-1<br>0, Immer

**SF-36 (visit 1, visit 7, 8** only if degree of pancreatic resection is not answered 'no pancreatoduodenectomy performed'**)**		
Variable	Type	Value
Datum des Ausfüllens<br>*If exact date not known, please skip this field and enter year and month (if known) below*	date	
Falls Datum unvollständig angegeben, bitte Monat eintragen	dropdown	01<br>02<br>03<br>04<br>…<br>11<br>12
Falls Datum unvollständig angegeben, bitte Jahr eintragen	integer	
1. Wie würden Sie Ihren Gesundheitszustand im Allgemeinen beschreiben?	radio	Ausgezeichnet<br>Sehr gut<br>Gut<br>Weniger gut<br>Schlecht
2. Im Vergleich zum vergangenen Jahr, wie würden Sie Ihren derzeitigen Gesundheitszustand beschreiben?	radio	Derzeit viel besser<br>Derzeit etwas besser<br>Etwa wie vor einem Jahr<br>Derzeit etwas schlechter<br>Derzeit viel schlechter
3.a. anstrengende Tätigkeiten	radio	Ja, stark eingeschränkt<br>Ja, etwas eingeschränkt<br>Nein, überhaupt nicht eingeschränkt
3.b. mittelschwere Tätigkeiten	radio	Ja, stark eingeschränkt<br>Ja, etwas eingeschränkt<br>Nein, überhaupt nicht eingeschränkt
3.c. Einkaufstaschen heben oder tragen	radio	Ja, stark eingeschränkt<br>Ja, etwas eingeschränkt<br>Nein, überhaupt nicht eingeschränkt

3.d. mehrere Treppenabsätze steigen	radio	Ja, stark eingeschränkt<br>Ja, etwas eingeschränkt<br>Nein, überhaupt nicht eingeschränkt
3.e. einen Treppenabsatz steigen	radio	Ja, stark eingeschränkt<br>Ja, etwas eingeschränkt<br>Nein, überhaupt nicht eingeschränkt
3.f. sich beugen, knien, bücken	radio	Ja, stark eingeschränkt<br>Ja, etwas eingeschränkt<br>Nein, überhaupt nicht eingeschränkt
3.g. mehr als einen Kilometer zu Fuß gehen	radio	Ja, stark eingeschränkt<br>Ja, etwas eingeschränkt<br>Nein, überhaupt nicht eingeschränkt
3.h. mehrere Straßenkreuzungen weit zu Fuß gehen	radio	Ja, stark eingeschränkt<br>Ja, etwas eingeschränkt<br>Nein, überhaupt nicht eingeschränkt
3.i. eine Straßenkreuzung weit zu Fuß gehen	radio	Ja, stark eingeschränkt<br>Ja, etwas eingeschränkt<br>Nein, überhaupt nicht eingeschränkt
3.j. sich baden oder anziehen	radio	Ja, stark eingeschränkt<br>Ja, etwas eingeschränkt<br>Nein, überhaupt nicht eingeschränkt
4.a Ich konnte nicht so lange wie üblich tätig sein	radio	Ja<br>Nein
4.b Ich habe weniger geschafft als ich wollt	radio	Ja<br>Nein
4.c Ich konnte nur bestimmte Dinge tun	radio	Ja<br>Nein
4.d Ich hatte Schwierigkeiten bei der Ausführung	radio	Ja<br>Nein
5.a Ich konnte nicht so lange wie üblich tätig sein	radio	Ja<br>Nein
5.b Ich habe weniger geschafft als ich wollte	radio	Ja<br>Nein
5.c Ich konnte nicht so sorgfältig wie üblich arbeiten	radio	Ja<br>Nein
6. Wie sehr haben Ihre körperliche Gesundheit oder seelischen Probleme in den vergangenen 4 Wochen Ihre normalen Kontakte zu Familienangehörigen, Freunden, Nachbarn oder zum Bekanntenkreis beeinträchtigt?	radio	Überhaupt nicht<br>Etwas<br>Mäßig<br>Ziemlich<br>Sehr
7. Wie stark waren Ihre Schmerzen in den vergangenen 4 Wochen?	radio	Keine Schmerzen<br>Sehr leicht<br>Leicht<br>Mäßig<br>Stark<br>Sehr stark

8. Inwieweit haben die Schmerzen Sie in den vergangenen 4 Wochen bei der Ausübung Ihrer Alltagstätigkeiten zu Hause und im Beruf behindert?	radio	Überhaupt nicht<br>Etwas<br>Mäßig<br>Ziemlich<br>Sehr
9.a … voller Schwung	radio	Immer<br>Meistens<br>Ziemlich oft<br>Manchmal<br>Selten<br>Nie
9.b … sehr nervös?	radio	Immer<br>Meistens<br>Ziemlich oft<br>Manchmal<br>Selten<br>Nie
9.c … so niedergeschlagen, dass Sie nichts aufheitern konnte?	radio	Immer<br>Meistens<br>Ziemlich oft<br>Manchmal<br>Selten<br>Nie
9.d … ruhig und gelassen?	radio	Immer<br>Meistens<br>Ziemlich oft<br>Manchmal<br>Selten<br>Nie
9.e … voller Energie?	radio	Immer<br>Meistens<br>Ziemlich oft<br>Manchmal<br>Selten<br>Nie
9.f ... entmutigt und traurig?	radio	Immer<br>Meistens<br>Ziemlich oft<br>Manchmal<br>Selten<br>Nie
9.g … erschöpft?	radio	Immer<br>Meistens<br>Ziemlich oft<br>Manchmal<br>Selten<br>Nie
9.h ... glücklich?	radio	Immer<br>Meistens<br>Ziemlich oft<br>Manchmal<br>Selten<br>Nie

9.i … müde?	radio	Immer<br>Meistens<br>Ziemlich oft<br>Manchmal<br>Selten<br>Nie
10. Wie häufig haben Ihre körperliche Gesundheit oder seelischen Probleme in den vergangenen 4 Wochen Ihre Kontakte zu anderen Menschen beeinträchtigt?	radio	Immer<br>Meistens<br>Manchmal<br>Selten<br>Nie
11.a Ich scheine etwas leichter als andere krank zu werden.	radio	Trifft ganz zu<br>Trifft weitgehend zu<br>Weiß nicht<br>Trifft weitgehend nicht zu<br>Trifft überhaupt nicht zu
11.b Ich bin genauso gesund wie alle anderen, die ich kenne.	radio	Trifft ganz zu<br>Trifft weitgehend zu<br>Weiß nicht<br>Trifft weitgehend nicht zu<br>Trifft überhaupt nicht zu
11.c Ich erwarte, dass meine Gesundheit nachlässt.	radio	Trifft ganz zu<br>Trifft weitgehend zu<br>Weiß nicht<br>Trifft weitgehend nicht zu<br>Trifft überhaupt nicht zu
11.d Ich erfreue mich ausgezeichneter Gesundheit.	radio	Trifft ganz zu<br>Trifft weitgehend zu<br>Weiß nicht<br>Trifft weitgehend nicht zu<br>Trifft überhaupt nicht zu
